# Work–Life Balance: Striking the Right Chords for Harmony

**DOI:** 10.3389/fped.2015.00122

**Published:** 2016-01-12

**Authors:** Kavita Morparia

**Affiliations:** ^1^Children’s National Medical Center, Washington, DC, USA

**Keywords:** work–life, burnout, professional, career satisfaction, women in science, work–family interference, pediatric intensive care, critical care

As a mother of two young children and a fellow in pediatric critical care, I was often asked the question: “how do you do it?” I certainly liked to think that I was balancing work and life appropriately. To be honest, more often than not there was simply no choice to be made about what the need of the hour was – the bare minimum at work and home accounted for every minute. When I took up additional research or childcare responsibilities, it translated into one thing: working even harder. Somehow, in addition to fulfilling the requirements of an academic fellowship, I managed to make time for doctors’ appointments, preschool events, and birthday parties. I was doing enough, but it was not enough to make me satisfied.

There is no objective way to measure what constitutes a good work–life balance. It is probably safe to assume, though that most of us have experienced a significant period of conflict between work and family at some point in time. Garcia et al. (1) reported a burnout level as high as 71% among Brazilian pediatric intensivists. While these data may not be reflective of prevalence in other countries, a rate of burnout as high as 50% was reported by Fields et al. (2) 20 years ago too. Burnout represents the far end of the spectrum of a chronically disrupted work–life balance, and these numbers should serve as a clarion call to us.

The Maslach Burnout Inventory Scale (3) focuses on three major domains: emotional exhaustion, depersonalization, and a sense of low personal accomplishment. Intensivists have consistently scored high in all three areas (4). While the presence of emotional exhaustion in intensivists makes intuitive sense, the other two components demand pause for thought: how does being so directly involved in patient care and working so closely with other team members coexist with feelings of depersonalization? How does one reconcile feelings of low accomplishment with saving lives?

As intensive care providers, we like to be in control of every situation and to be proactive with our choices. We like to do it all and have it all, both at life and work. Just as anything less than perfect is unacceptable at work, so too settling for less than the best for our children and families will not do. In this pursuit of perfection, the inevitable victim is our personal time. As I went through training, the cumulative burden of the many moments when I lacked the flexibility of doing something that I really wanted to started adding up. As weeks and months of training started blurring together, I felt an experiential loss. It is said that life is in the details, and in the daily grind, I was missing out on the texture of things, the richness of context. Spending time with friends and loved ones serves more than a purely social purpose. Music and books help us to briefly step outside the reality of our own narrow existences. Perspective can only be gained from a distance. Amid death and suffering, we need to acknowledge our emotions and process them in a timely manner. When harnessed appropriately, these same emotions can enhance learning and situational memory. When I was talking to a concerned parent, but really thinking about the critically ill child in the next room, I could not shake the feeling that I was somehow short-changing the parent. I was too busy to know my patients on a personal level anymore. Lack of time to research clinical dilemmas as they occurred left me with a nagging sense of a job not completed, as well as worry that I was passing up optimal learning opportunities. Accrual of information can occur semi-automatically; active learning requires concerted study, a re-examination of all prior knowledge to reconcile with new evidence. Accordingly, treating patients per guidelines and protocols was easy; it was harder to have the force of conviction behind my decisions when I had not spent enough time thinking about them. Creativity, a requirement for cognitive growth, cannot be cultivated when time is broken down into bite-sized pieces. Sitting in the comfort of my home, sipping on a cup of coffee while I type this, I can rationalize that I was doing what was necessary and required of me. It was plenty. In the moment, however, I felt like I was running a marathon, when I really wanted to cover the distance by alternately sprinting and resting.

Thankfully, I had less control over my now 4-year-old son, who was innately better than me at seizing the moment and taking me along for the ride. If I was required to do nothing but serve as a cushion for a whole hour, I had no choice but to comply. We took walks together, baked cookies, rode the miniature train. I might have elected to do something more “high-yield” if I had the time to plan ahead – register for a gym class or swim lessons, set play dates. In retrospect, I am glad I did not; I dearly cherish the time spent with him.

Chittenden and Ritchie (5), in their excellent review, describe several strategies for achieving a good work–life balance. One of these is “timeshifting”: the ability to really slow down and relax during downtime. This approach often requires a deliberate mindfulness of our thoughts and controlling the urge to work during periods set aside for rest. Setting goals and revisiting career plans with trusted mentors at regular intervals is worthwhile. It is crucial to devote time for reflection to enable incorporation of changing priorities and values into future goals, as life circumstances continue to change. Learning to live in the moment is something our children can teach us. Seeking feedback can help us uncover our own blind spots. Importantly for intensivists, being realistic with expectations and replacing self-criticism with positive change can go a long way.

Without a doubt, critical care medicine can be mentally, physically, and emotionally draining, but it can also be immensely rewarding. As intensivists, we are privileged to help families through what will probably be the most challenging time of their lives. We get to see the fruits of our labor in the here and now, when we successfully resuscitate a child or perform a lifesaving procedure. We are witness every day to the remarkable resilience of children and the grace with which parents bear their impossible burdens. We are looked upon as leaders of a large team. Yes, work hours are long, but time away from the unit is uninterrupted by consultations and clinics. Most importantly, we are doing what appeals most to us. Work itself is a large part of our identity, self-worth, and purpose in life. It is natural to see our own problems as trivial and neglect our own well-being. The biggest lesson I have learnt from my experience thus far is a simple one: “Make time to do what makes you happy.”

When I reframed my goal to achieve a positive “work–life-self” balance, I become a better doctor and mother, and a happier person. The “self” had to be fitted in somehow, but I discovered how elastic time could be and that only real rest led to real productivity. It is fitting to end with these lines by Matthew Arnold, a reassurance that every effort counts to making us better physicians, whether or not we can see it as such at the time.

“With aching hands and bleeding feet

We dig and heap, lay stone on stone:

We bear the burden and the heat

Of the long day, and wish ‘twere done.

Not till the hours of light return,

All we have done do we discern.”

## Author Contributions

KM was responsible for writing this opinion piece.

## Conflict of Interest Statement

The author declares that the research was conducted in the absence of any commercial or financial relationships that could be construed as a potential conflict of interest.
